# Importance and Limits of Ischemia in Renal Partial Surgery: Experimental and Clinical Research

**DOI:** 10.1155/2008/102461

**Published:** 2008-07-15

**Authors:** Fernando P. Secin

**Affiliations:** Urology Section, CEMIC University Hospital, Buenos Aires C1431FWO, Argentina

## Abstract

*Introduction*. The objective is to determine the clinical and experimental evidences of the renal responses to warm and cold ischemia, kidney tolerability, and available practical techniques of protecting the kidney during nephron-sparing surgery. *Materials and methods*. Review of the English and non-English literature using MEDLINE, MD Consult, and urology textbooks. *Results and discussion*. There are three main mechanisms of ischemic renal injury, including persistent vasoconstriction with an abnormal endothelial cell compensatory response, tubular obstruction with backflow of urine, and reperfusion injury. Controversy persists on the maximal kidney tolerability to warm ischemia (WI), which can be influenced by surgical technique, patient age, presence of collateral vascularization, indemnity of the arterial bed, and so forth. *Conclusions*. When WI time is expected to exceed from 20 to 30 minutes, especially in patients whose baseline medical characteristics put them at potentially higher, though unproven, risks of ischemic damage, local renal hypothermia should be used.

## 1. INTRODUCTION

Nephron-sparing surgery in the oncologic setting entails complete local resection of a renal tumor while leaving the largest possible amount of normal functioning parenchyma in the involved kidney. Different surgical techniques can be employed for performing partial nephrectomy, but all of them require adherence to basic principles of early vascular control, avoidance of ischemic renal damage with complete tumor excision with free margins, precise closure of the collecting system, careful hemostasis, and closure with or without tamponading of the renal defect with adjacent fat, fascia, or any available artificial sealant [1, 2].

Observance of all these principles is extremely important, however, prevention of ischemic renal damage is a key to the final success of the procedure. Ischemia is the leading cause of postoperative acute and chronic renal failure in patients undergoing nephron sparing surgery, for which no specific medical treatment modality has been established to date.

By the same token, surgeons need to apply transitory occlusion of the renal artery as it not only diminishes intraoperative parenchymal bleeding but also improves visualization and facilitates access to intrarenal structures by causing the kidney to contract and by reducing renal tissue fullness. Surgeons performing this approach require an understanding of renal responses to warm ischemia (WI) and available methods of protecting the kidney when the period of arterial occlusion exceeds normal parenchyma tolerability [3].

In order to decrease the exposure of the spared parenchyma to ischemia, the surgeon should have a complete preoperative and intraoperative assessment of the relationship of the tumor and its vascular supply to the collecting system and adjacent normal renal parenchyma [4–6].

There is no question that the less the better, whenever the philosophy to preserve as much functioning renal tissue as possible is followed. This manuscript seeks to determine the clinical and experimental evidences of the renal responses to warm and cold ischemia, kidney tolerability, and available practical techniques of protecting the kidney when the period of arterial occlusion surpasses that which may be safely tolerated during renal nephron sparing surgery.

## 2. MATERIAL AND METHODS

Biomedical and related databases were queried including MEDLINE, MD Consult, and urology textbooks. Manuscripts and library archives were retrieved from the Nathan Cummings Center, Memorial Sloan-Kettering Cancer Center, NY, USA.

A Medline search in combination with additional references of non-Medline-indexed journals included the following key words: “nephron-sparing surgery,” “partial nephrectomy,” “warm ischemia and kidney,” and “ischemia time and kidney,” as well as links to related articles. Non-English articles and letters to editors were reviewed as well. These references formed the basis of the article. Following selection and deletion based on relevance of the subject and importance of the studies, a library of 115 references remained.

## 3. RESULTS AND DISCUSSION

### 3.1. Intraoperative renal ischemia: pathophysiology of injury

In recent years, there have been significant insights into the pathophysiologic process of renal ischemia [7, 8]. Ischemic insult to the kidney often results in damage to cells of nephron and renal vasculature. Cells are lost through the processes of necrosis and apoptosis, inevitably leading to renal failure. Renal failure is characterized by a decline in glomerular filtration rate, retention of nitrogenous waste products, perturbation of extra cellular fluid volume, and electrolyte and acid-base homeostasis. Renal failure is only diagnosed when these pathophysiologic perturbations are advanced enough to manifest biochemical abnormalities in the blood. The pathophysiologic response to cell death dictates the prevailing level of renal functional impairment [9]. Therefore, a clear understanding of the extent of post ischemic kidney damage and associated inflammation is needed to prevent this hitherto intractable condition, which will ultimately impact on overall survival [10].

For understanding and didactic purposes, three interrelated main mechanisms through which ischemia damages the kidney are herein described based on a recent review by Abuelo [7]. One mechanism is merely vascular, caused by persistent vasoconstriction and an abnormal response of endothelial cells to compensatory means. The second is obstructive, where soughed tubular epithelial cells and brush-border-membrane debris form casts that obstruct tubules, and glomerular filtrate leaks from the tubular lumen across denuded tubular walls into capillaries and the circulation (back-leak) causing a reduction in the “effective” GFR, where the latter is defined as the rate at which filtrate is delivered into final urine. The third has to do with reperfusion injury after blood flow is restored [7, 11].

#### 3.1.1. Vascular mechanism

Both animal and human studies have found that a multi-inflammatory response is involved in ischemia/reperfusion injury of the kidney [12]. The inflammatory reaction incurred after an ischemic insult precipitates more damage to the tissue and impedes intrarenal blood flow caused by vasoconstriction and vascular congestion, leading to a vicious cycle [13].

This damage mainly takes place in endothelial cells of the peritubular capillaries, especially in the outer medulla, which is marginally oxygenated under normal circumstances. This oxidant injury, together with a shift in the balance of vasoactive substances toward vasoconstrictors such as endothelin, results in vasoconstriction, congestion, hypoperfusion, and expression of adhesion molecules. The expression of adhesion molecules, in turn, initiates leukocyte infiltration, augmented by proinflammatory and chemotactic cytokines generated by ischemic tubular cells [7].

Inciting stimuli induce kidney macrophages and probably renal parenchymal cells to release inflammatory cytokines, such as tumor necrosis factor-*α* (TNF-*α*) and interleukin-1 (IL-1). TNF-*α* and IL-1 promote renal parenchymal damage by directly inducing apoptosis in epithelial cells, recruitment of neutrophils that release reactive oxygen metabolites and proteases, and up regulating adhesion receptors on endothelial cells and leukocytes [14, 15]. These cytokines also stimulate renal cortical epithelial cells to release the chemoattractant interleukin-8 [16, 17]. The arrival of additional leukocytes obstructs the microcirculation and releases more cytotoxic cytokines, reactive oxygen species, and proteolytic enzymes, which damage the tubular cells [7].

Endothelial injury results in cell swelling and enhanced expression of cell adhesion molecules. This, together with leukocyte activation, leads to enhanced leukocyte-endothelial cell interactions, which can promote injury and swelling of the endothelial cell. Endothelial swelling contributes to the production of local factors promoting vasoconstriction and adds to the effects of vasoconstriction and tubule cell metabolism by physically impeding blood flow, perpetuating that vicious cycle [18].

Heterogeneity of intrarenal blood flow contributes to the pathophysiology of ischemic renal failure. An imbalance between the vasodilator nitric oxide and the vasoconstrictor endothelin impairs medullary blood flow, especially in the outer medulla, where tubules have high oxygen requirements, resulting in cellular injury due to a mismatch between oxygen delivery and demand. Endothelial activation and injury together with increased leukocyte-endothelial cell interactions and activation of coagulation pathways may have a greater effect on outer medullary ischemia than arteriolar vasoconstriction, as there can be markedly impaired oxygen delivery to the outer medulla despite adequate renal blood flow [18].

The arteriolar response to vasoactive substances can also be altered during endothelial injury. The basal tone of arterioles is increased in post ischemic kidneys as well as their reactivity to vasoconstrictive agents. These arterioles also have decreased vasodilatory responses compared with arterioles from normal kidneys. Alterations in local levels of vasoconstrictors (angiotensin II, thromboxane A_2_, leukotrienes, adenosine, endothelin-1) have been implicated in abnormal vascular tone [19]. Angiotensin II seems to play a key role by activating endothelin B or prostaglandin H_2_-thromboxane A_2_ receptors. Systemic endothelin-1 levels increase with ischemia, and administration of antiendothelin antibodies or endothelin receptor antagonists has been reported to protect against ischemia-reperfusion injury [20]. Saralasin, an angiotensin II receptor antagonist, could also attenuate angiotensin II vasoconstricting effect [21]. Nitric oxide, an endothelial-derived relaxing factor, plays a theoretical protective role against ischemic renal injury, by means of its vasodilatory effect and by decreasing endothelin expression and secretion in the vascular endothelium. Of interest, endothelial nitric oxide synthase is inhibited during endothelial injury [22]. A combination therapy consisting of 5-aminoimidazole-4-carboxamide-1-beta-D-ribonucleoside (AICAR) and N-acetyl cysteine (NAC), drugs that inhibit the induction of proinflammatory cytokines and nitric oxide synthase, and block tumor necrosis factor-alpha induced apoptotic cell death, has shown to attenuate ischemia-reperfusion injury in a canine model of autologous renal transplantation [23]. Early studies showed no conclusive evidence that vasodilators (such as diltiazem or dopamine) or other compounds have any clinical utility in either preventing or treating ischemic renal failure in humans thus far [24–26]. More recently, however, the highly selective dopamine type 1 agonist fenoldopam mesylate [27] and the antianginal medication trimetazidine [28] appeared to aid in restoring renal function to baseline values in patients with prolonged WI time. Further research is needed.

#### 3.1.2. Obstructive mechanism

Normally, the cells are bathed in an extra cellular solution high in sodium and low in potassium. This ratio is maintained by a sodium pump (Na^+^-K + ATPase pump) which uses much of the adenosine triphosphate (ATP) energy derived from oxidative phosphorylation. ATP is required for the cellular sodium pump to maintain a high intracellular concentration of potassium and a low concentration of sodium. The sodium pump effectively makes Na^+^ an impermeant outside the cell that counteracts the colloidal osmotic pressure derived from intracellular proteins and other anions [29].

The ischemic insult causes a failure of oxidative phosphorylation and ATP depletion, leading to malfunctioning of the sodium pump. When the sodium pump is impaired, sodium chloride and water passively diffuse into the cells, resulting in cellular swelling and the “no-reflow” phenomenon after renal reperfusion. Cellular potassium and magnesium are lost, calcium is gained, anaerobic glycolysis and acidosis occur, and lysosomal enzymes are activated. This results in cell death. During reperfusion, hypoxanthine, a product of ATP degradation, is oxidized to xanthine with the formation of free radicals that cause further cell damage [29]. (See later.)

As mentioned, the mechanism whereby ischemia and oxygen depletion injure tubular cells starts with ATP depletion, which activates a number of critical alterations in metabolism, causing cytoskeletal disruption and loss of those properties that normally render the tubule cell monolayer impermeable to certain components of filtrate. Cytoskeletal disruption causes not only loss of brush-border microvilli and cell junctions but also mislocation of integrins and the sodium pump from the basal surface to the apical surface.

In addition, impaired sodium reabsorption by injured tubular epithelial cells increases the sodium concentration in the tubular lumen. The increased intratubular sodium concentration polymerizes Tamm-Horsfall protein, which is normally secreted by the loop of Henle, forming a gel and contributing to cast formation. As a result, brush-border membranes and cells slough obstruct tubules downstream. As mentioned before, these debris form casts that obstruct tubules, and glomerular filtrate leaks from the tubular lumen across denuded tubular walls into capillaries and the circulation (back-leak) causing a reduction in the “effective” GFR. ATP depletion also activates harmful proteases and phospholipases, which, with reperfusion, cause oxidant injury to tubular cells, the so-called reperfusion injury [7].

#### 3.1.3. Reperfusion injury

WI insult followed by restoration of blood flow to the ischemic tissue frequently results in a secondary reperfusion injury. Despite WI causing significant renal dysfunction, reperfusion injury has been shown to be as damaging or even more detrimental than renal ischemia itself, producing an inflammatory response that worsens local kidney damage and leads to a systemic insult [30, 31].

The reperfusion injury can be mediated by several mechanisms including the generation of reactive oxygen species, cellular derangement, microvessel congestion and compression, polimorphonuclear (PMN)-mediated damage, and hypercoagulation. Reperfusion with the resulting reintroduction of molecular oxygen of constricted microvessels leads to congestion and red cell trapping. This vascular effect can reduce renal blood flow by as much as 50% [32].

During the reperfusion period, superoxide production in the kidney is markedly enhanced by the transformation of xanthine dehydrogenase to xanthine oxidase and the increase in free electrons in mitochondria, prostaglandin H, and lipoxygenase with the coexistence of NAD(P)H and infiltrated neutrophils. Superoxide raises the following chain reactions, producing hydroxyl radicals or other reactive oxygen species (ROSs), or interacts with nitric oxide (NO), which is produced by macrophage inducible NO synthase, generating a highly toxic radical peroxynitrite. These ROS and NO derived species consume tissue antioxidants and decrease organ reducing activity [33].

The exact magnitude of reperfusion injury is still unclear. Some authors state that the role of free radicals mediated injury in kidneys may not be as significant as in other organs given the low relative activity of renal xanthine oxidase compared with the high endogenous activity of superoxide dismutase [29].

Notwithstanding, nicaraven (N,N9-propylenebisnicotinamide), a drug that may actively trap free radicals and prevent vascular constriction due to lipid peroxide [34] and edaravone (3-methyl-1-phenyl-2-pyrazolin-5-one, MCI-186), a synthetic free radical scavenger, have shown in vitro experiments to protect endothelial cells against ischemic injury in different organs, including ischemically damaged kidneys [35, 36]. Clinical studies are eagerly awaited.

## 4. FOR HOWLONG CAN THE KIDNEY TOLERATE WARM ISCHEMIA?

Despite several animal studies [37–39] and clinical reports [40, 41] demonstrating kidney tolerance to warm ischemia times beyond 30 minutes, concern still remains regarding the potential for full-renal function recovery after this time period [42]. The stoic 30-minute cutoff has been questioned by some authors [43] on the grounds that kidneys harvested from nonheart beating donors (NHBDs) have shown favorable recovery of renal function in transplanted kidneys that sustained warm ischemia times well over 30 minutes [44–46]. Nishikido et al. [45] found that the risk factors affecting significant *graft loss* were WI time more than 20 minutes, donor age above 50 years, and donor serum creatinine at admission above 1.0 mg/dL. Today, most nonheart beating donor programs currently exclude those donors with a WI time exceeding 40 minutes [45, 47–49].

Although laparoscopic surgeons are gaining further experience and are more ambitious to perform partial nephrectomy for larger and deeper tumors, the 30-minute cutoff still remains the accepted safe limit time beyond which irreversible kidney damage occurs in the absence of renal cooling [50–52].

Although early observations in dog models showed that there may be substantial variation in kidney tolerance up to two or three hours of ischemia [53] there is no doubt that the extent of renal damage after transitory arterial occlusion exclusively depends on the duration of the ischemic insult [25, 54, 55]. The literature also demonstrates that, even within a tolerable period of WI, the longer the WI time the longer it takes for the kidney to recover (or approach) its preoperative function [55]. Notwithstanding, the maximum tolerable limit of renal warm ischemia time that can render complete function recovery remains to be established in humans.

The study by Ward [56] is commonly cited by opinion leaders to state a maximum 30-minute tolerance of the kidney to WI. These authors showed in dogs that warm ischemic intervals of up to 30 minutes can be sustained with eventual full recovery of renal function. However, this study was not strictly designed to establish the most accurate length of time a kidney would be able to sustain reversible damage following ischemic injury. What the authors actually concluded was that no additional protection to ischemia could be gained by cooling below 15 degrees. Thus, they recommended 15 degrees as the optimum temperature for use in clinical renal hypothermia.

Research in rats, pigs, and monkeys has also been conducted by other investigators. Laven et al. [38] found renal resilience to WI beyond the traditionally accepted 30 minutes in a solitary kidney pig model. Prolonged renal WI time increased the incidence of renal dysfunction during the initial 72 hours after the ischemic insult. However, by 2 weeks after the WI insult renal function returned to baseline in the 30, 60, and 90-minute WI groups. However, the same study group found that prolonged WI time of 120 minutes produced significant loss of renal function and mortality [43].

Martin et al. [57] proved potential kidney WI tolerability of up to 35 minutes in a single kidney monkey model.

Haisch et al. [58] studies in dog models suggested that the window of reversible WI injury could be as long as 2 hours after the insult.

The question remains whether findings in animal studies can be extrapolated to humans. One limitation has to do with a reliable method to differentiate between ischemic injury and the loss of renal volume secondary to tumor excision. The ideal method to evaluate residual kidney function in the operated kidney is still undefined. While most authors use serum creatinine assay or 99mtechnetium-labeled mercaptoacetyl triglycine (MAG3) renal scintigraphy with split renal function, others, like Abukora et al. [59] proposed estimation of parenchymal transit time (PTT) as a good indicator of ischemic injury. Transit time is the time that a tracer remains within the kidney or within a part of the kidney. However, the international consensus committee on renal transit time, from the subcommittee of the International Scientific Committee of Radionuclides in Nephrourology, recently concluded that the value of delayed transit remains controversial, and the committee recommended further research [60].

Bhayani et al. [40] evaluated 118 patients, with a single, unilateral, sporadic renal tumor, and normal contralateral kidney, who underwent laparoscopic partial nephrectomy (LPN) to assess the effect of variable durations of WI on long-term renal function. Patients were divided into 3 groups based on WI time: group 1, no renal occlusion (*n* = 42), group 2, WI < 30 minutes (*n* = 48), and group 3, WI > 30 minutes (*n* = 28). At a median followup of 28 months (minimum followup of 6 months) median creatinine had not statistically increased postoperatively and none of the 118 patients progressed to renal insufficiency or required dialysis after LPN. The authors concluded that WI time up to 55 minutes did not significantly influence long-term renal function after LPN. A main limitation of this study has to do with the fact that all patients had a normal contralateral kidney so that 6 months postoperatively creatinine values could have reflected contralateral kidney function.

A similar study has been conducted by Shekarriz et al. [61] on a substantially lower number of patients (*n* = 17); however, the authors assessed kidney function using 99technetium labeled diethylenetetraminepentaacetic acid scan renal scan with differential function 1 month before and 3 months after surgery in all patients. The authors found that all their patients preserved adequate renal function in the affected kidney following temporary hilar clamping of up to 44 minutes. (The mean WI time was 22.5 minutes.)

In line with this author, Kane et al. [62] showed that temporary arterial occlusion did not appear to affect short-term renal function (mean followup: 130 days) in a series of laparoscopic partial nephrectomies (LPNs) with a mean WI of 43 minutes (range: 25–65 minutes).

Desai et al. [50] retrospectively assessed the effect of WI on renal function after LPN for tumor, and evaluated the influence of various risk factors on renal function in 179 patients under WI conditions. No kidney was lost because of ischemic sequelae with clamping of the renal artery and vein of up to 55 minutes. The mean WI time was 31 minutes. Nonetheless, the authors concluded that advancing age and pre-existing azotaemia increased the risk of renal dysfunction after LPN, especially when the warm ischemia exceeded 30 minutes.

In contrast, Kondo et al. [63] found that patient age did not influence residual function in patients undergoing partial nephrectomy, while tumor size was the only significant factor that inversely correlated with the relative 99technetium labeled dimercaptosuccinic acid (DMSA) uptake.

Porpiglia et al. [52] assessed kidney damage in 18 patients 1 year after LPN with a WI time between 31 and 60 minutes. The authors evaluated the contribution of the operated kidney to the overall renal function by radionuclide scintigraphy with 99mTc-MAG3. They observed that there was an initial significant drop of approximately 11% in the operated kidney's contribution to overall function, followed by a constant and progressive recovery that never reached the preoperative value (42.8% at 1 year versus 48.3% before surgery). The authors stated by logistic regression analysis that the loss of function of the operated kidney depended mostly on the WI time and less importantly on the maximum thickness of resected healthy parenchyma. Unfortunately, the full regression model that included 6 variables to predict an event in only 18 patients is not shown in the manuscript.

Recently, Thompson et al. [42] made a retrospective review of 537 patients with solitary kidneys who underwent open nephron sparing surgery by more than 20 different surgeons from both the Cleveland Clinic, Ohio, USA, and Mayo Clinic, Minn, USA, to evaluate the renal effects of vascular clamping in patients with solitary kidneys. After adjusting for tumor complexity and tumor size, the author found in a subsequent analysis [64] that patients with more than 20 minutes of WI were significantly more likely to have acute renal failure (24% versus 6%, p 0.002) compared to those requiring less than 20 minutes, and this risk remained significant even after adjusting for tumor size (odds ratio 3.4, p 0.025). Additionally, patients with more than 20 minutes of WI were significantly more likely to progress to chronic renal failure (odds ratio 2.9, p 0.008) and were more than 4 times more likely to experience an increase in creatinine postoperatively of greater than 0.5 mg/dL (odds ratio 4.3, p 0.001) compared to those requiring less than 20 minutes of WI. After adjusting for tumor size, the risk of chronic renal failure (odds ratio 2.6, p 0.03) and an increase in creatinine of greater than 0.5 mg/dL (odds ratio 4.6, p 0.002) remained statistically significant if more than 20 minutes of WI were needed. The authors concluded that WI should be restricted to less than 20 minutes when technically feasible, especially in patients with solitary kidneys.

## 5. WHAT ARE THE FACTORS AFFECTING TOLERANCE TOWARM ISCHEMIA?

It often goes without saying that there may be individual variation to WI tolerance. Baldwin et al. [37] observed that some of the 16 solitary porcine kidneys showed a rapid return to the dark red color, and other animals demonstrated minimal color change during the several minutes following complete hilar clamp removal, despite all of them receiving similar surgical technique and ischemia time. Having acknowledged the potential for individual variation, there may be other multiple factors that can affect tolerance to WI which are herein described.

It has been suggested that patients with solitary kidneys might safely tolerate longer periods of ischemia than patients with both kidneys as the result of development of a collateral vascular supply; [65–67] however, the presence of vascular collateralization secondary to vascular occlusive disease, [68] or yet other clinical entities like hypertension, [69] should warn the surgeon for the possibility of a kidney less resistant to WI injury for the likely presence of panvascular disease and or occult chronic renal insufficiency.

Another factor that can impact ischemic damage is the method employed to achieve vascular control of the kidney. When technically possible, depending on the size and location of the tumor, it is helpful to leave the renal vein patent throughout the operation. This measure has been proven to decrease intraoperative renal ischemia and, by allowing venous backbleeding, facilitates hemostasis by enabling identification of small, transected renal veins [1–3, 5].

Animal studies have shown that functional impairment is least when the renal artery *alone* is occluded. Although some authors found no difference [70] simultaneous occlusion of the renal artery and vein for an equivalent time interval is more damaging because it prevents, as mentioned, retrograde perfusion of the kidney through the renal vein and may also produce venous congestion of the kidney [2, 3, 71–73]. However, this benefit may not be observed in patients undergoing LRP since the pressure of the pneumoperitoneum may cause partial occlusion of the renal vein, thus, negating the advantage of renal artery clamping only [72].

Intermittent clamping of the renal artery with short periods of recirculation may also be more damaging than continuous arterial occlusion, possibly because of the release and trapping of damaging vasoconstrictor agents within the kidney [39, 55, 71, 74–77].

Manual (or instrumental) compression of the kidney parenchyma to control intraoperative hemorrhage (as an alternative to clamping of the pedicle) has the theoretical advantages of avoiding WI of the normal parenchyma while allowing the surgeon to operate in an almost bloodless field, something that could be particularly useful in peripherically located tumors. Although animal studies have shown that the use of renal parenchyma compression may be more deleterious than simple arterial occlusion [71, 76], this technique has been recently “resuscitated” by some authors both in the open kidney surgery [78–82] and in the laparoscopic setting [83].

When the surgeon anticipates a WI time exceeding the “classical” 30 minutes, local renal hypothermia is used to protect against ischemic renal injury. Hypothermia has been the most effective and universally used means of protecting the kidney from the ischemic insult. Hypothermia reduces basal cell metabolism, energy-dependent metabolic activity of the cortical cells, with a resultant decrease in both the consumption of oxygen and ATP [84–86].

There are multiple ways of achieving hypothermia. Surrounding the fully mobilized kidney with crushed ice (ice slush) is the most frequently used technique because of its ease and simplicity [87, 88]. When using ice slush to reduce kidney temperature, it is recommended to keep the entire kidney covered with ice for 10 to 15 minutes immediately after occluding the renal artery and before commencing the resection of the tumor in order to allow core renal temperature to decrease to approximately 20 degrees centigrade or less [2]. Mannitol, with or without the addition of furosemide, should be administered intravenously 5 to 15 minutes before renal arterial clamping as it increases renal plasma flow, decreases intrarenal vascular resistance and intracellular edema, and promotes an osmotic diuresis when renal circulation is restored [89]. Regular use of heparin to prevent intrarenal vascular thrombosis has not been found to be useful [2, 3, 56].

Other methods than the use of ice slush to achieve renal hypothermia have also being explored, including application of ice-slurry [90, 91], antegrade perfusion of the renal artery either via preoperative renal artery catheterization [92] or via intraoperative renal artery cannulation [93], retrograde perfusion of the collecting system with cold solutions [94, 95] or near-freezing saline irrigation delivered with a standard irrigator aspirator [96] among others, some of them particularly used in the laparoscopic setting. Very few studies compared kidney cooling techniques; [97–100] however, hypothermia by properly applying ice to the renal surface seems to be equivalent to hypothermia by perfusion [98]. Perfusion of the kidney with a cold solution instilled via the renal artery not only may have a theoretic risk of tumor dissemination, but also requires participation of an intervention radiology team to perform preoperative renal artery catheterization, adding complexity and risks of potential complications to the procedure [3]. On the contrary, continuous renal perfusion might have the advantage of providing a more homogeneous and effective hypothermia for a more extended period of time [99, 100]. It is generally accepted, founded on data extrapolated from the kidney stone literature, that adequate hypothermia provides up to 2 to 3 hours of renal protection from circulatory arrest [99, 101–104].

Needless to say, generous preoperative and intraoperative hydration, prevention of intraoperative hypotension, avoidance of unnecessary manipulation or traction on the renal artery as well as the aforementioned administration of mannitol are necessary to keep the kidney adequately perfused before and after the ischemic insult.

Ischemic preconditioning (IP) has emerged as a powerful method of ameliorating ischemia/reperfusion injury not only the myocardium (as initially described) [105] but also to other organs, including kidney. IP is a physiologic phenomenon by which cells develop defense strategies to allow them survive in a hypoxic environment. The original IP hypothesis stated that multiple brief ischemic episodes applied to an organ would actually protect it (originally the myocardium) during a subsequent sustained ischemic insult so that, in effect, ischemia could be exploited to protect that organ (originally the heart) from ischemic injury [105]. The “preconditioned” cells would become more tolerant to ischemia by adjusting its energy balance to a new, lower steady-state equilibrium. Specifically, preconditioned tissues exhibit reduced energy requirements, altered energy metabolism, better electrolyte homeostasis and genetic reorganization, giving rise to the concept of “ischemia tolerance.” IP also induces “reperfusion tolerance” with less reactive oxygen species and activated neutrophils released, reduced apoptosis and better microcirculatory perfusion compared to not preconditioned tissue. Systemic reperfusion injury is also diminished by preconditioning [31]. A review by Pasupathy and Homer-Vanniasinkam [31] showed that IP utilizes endogenous mechanisms in skeletal muscle, liver, lung, kidney, intestine, and brain in animal models to convey varying degrees of protection from reperfusion injury. To date, there are few human studies, but some reports suggest that human liver, lung, and skeletal muscle acquire similar protection after IP. IP is ubiquitous but more research is required to fully translate these findings to the clinical arena.

Some authors propose that during laparoscopy, the increase of intra-abdominal pressure due to the pneumoperitoneum may create an IP-like situation that might increase kidney tolerance to subsequent WI and reduce tissue injury [106–110]. For this reason, it might theoretically be possible to increase WI time during LPN, compared to open surgery, something which is still very far from being demonstrated [30, 109–111].

In contrast, other studies expressed some concern about the potential harm of pneumoperitoneum and increased intra-abdominal pressure (IAP) on kidney function. Several experimental animal studies have investigated the effect of pneumoperitoneum on renal function. While some authors demonstrated that increased IAP by insufflation of CO2 gas resulted in decreased renal blood flow that may lead to ischemia and subsequent decreased glomerular filtration rate [112], others denied such effect [37, 113].

Kirsch et al. [112] showed a decrease in urine output and GFR with increasing IAP. A pneumoperitoneum of 15 mmHg for 4 hours resulted in a decrease in renal blood flow to 70% of baseline. Even IAPs of 4 and 10 mmHg resulted in a reduction of the renal circulation of 34% and 41%, respectively. Although, the decreased urinary output during prolonged IAP greater than or equal to 15 mmHg in the animal model was associated with a corresponding decrease in renal vein flow, it did not appear to be associated with any permanent renal derangement nor any transient histological changes [114]. After the release of the pneumoperitoneum or pneumoretroperitoneum, the renal function and urine output return to normal with no long-term sequelae, even in patients with pre-existing renal disease [115].

Lind et al. [113] found that WI time of 20 minutes did not impair graft function and histomorphology during 1 year of followup after renal transplantation in a syngeneic rat model. Most important, WI in combination with pneumoperitoneum did not result in an additive negative effect on long-term graft function.

In addition, Baldwin et al. [37] observed that temporary serum creatinine elevation evident after 60 and 90 minutes of ischemia normalized within 7 days in 16 farm pigs which had been nephrectomized 14 days prior to the laparoscopically applied ischemic insult. No difference from the controls was noted in those pigs receiving 30 minutes of ischemia during the laparoscopic procedure. Of note, insufflation had been maintained for 150 minutes at 15 mmHg in all animals. Those findings suggested that in laparoscopic renal surgery, WI times of up to 90 minutes (and a pneumoperitoneum of up to 150 minutes) might be well tolerated and followed by complete renal recovery. The reader is referred to the excellent review by Dunn and McDougall [115] for further information on the impact of pneumoperitoneum on renal physiology.

## 6. CONCLUSIONS

The maximal duration of WI allowable before the onset of irreversible renal damage continues to be a topic of debate, irrespective of the surgical approach. In addition, there seems to be variation among patients, possibly related to surgical technique, patient age, presence of collateral vascularization, and indemnity of the arterial bed, among others. Unfortunately, no method exists for predicting preoperatively or intraoperative monitoring for renal injury. Surgeons should exert extreme efforts to keep warm ischemia time as short as possible. When WI time is expected to exceed from 20 to 30 minutes, specially in patients whose baseline medical characteristics put them at potentially higher, though unproven, risks of ischemic damage, the time-tested way around this time limit has been renal hypothermia, regardless of what the time limit may exactly be.
